# Supplementary material for: Improved Synthesis of Phosphoramidite-Protected N6-Methyladenosine via BOP-Mediated SNAr Reaction
Source: Molecules. 2020 Dec 31;26(1):147. doi: 10.3390/molecules26010147 (PMC7796277; doi:10.3390/molecules26010147)

Supplementary Information

Improved synthesis of phosphoramidite protected N^6-methyladenosine via BOP mediated S_NAr reaction

Shifali Shishodia[1,2] and Christopher J. Schofield[1,*]

[1] Department of Chemistry, Chemistry Research Laboratory, University of Oxford, 12 Mansfield Road, Oxford, OX1 3TA, United Kingdom.

[2] Present address: Department of Biochemistry, Medical College of Wisconsin, Milwaukee, Wisconsin 53226, United States

* Email: christopher.schofield@chem.ox.ac.uk

3´,5´-*O*-(Di-*tert*-butyl)silyl-2´-*O*-dimethyl(*tert*-butyl)silylinosine (2)

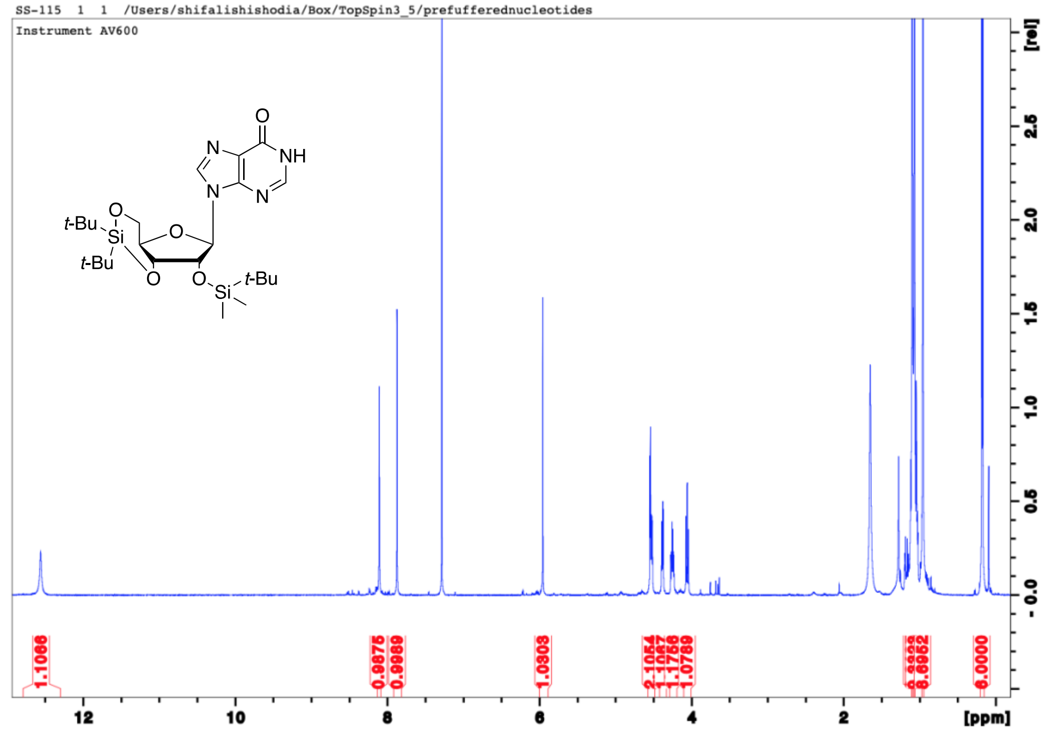

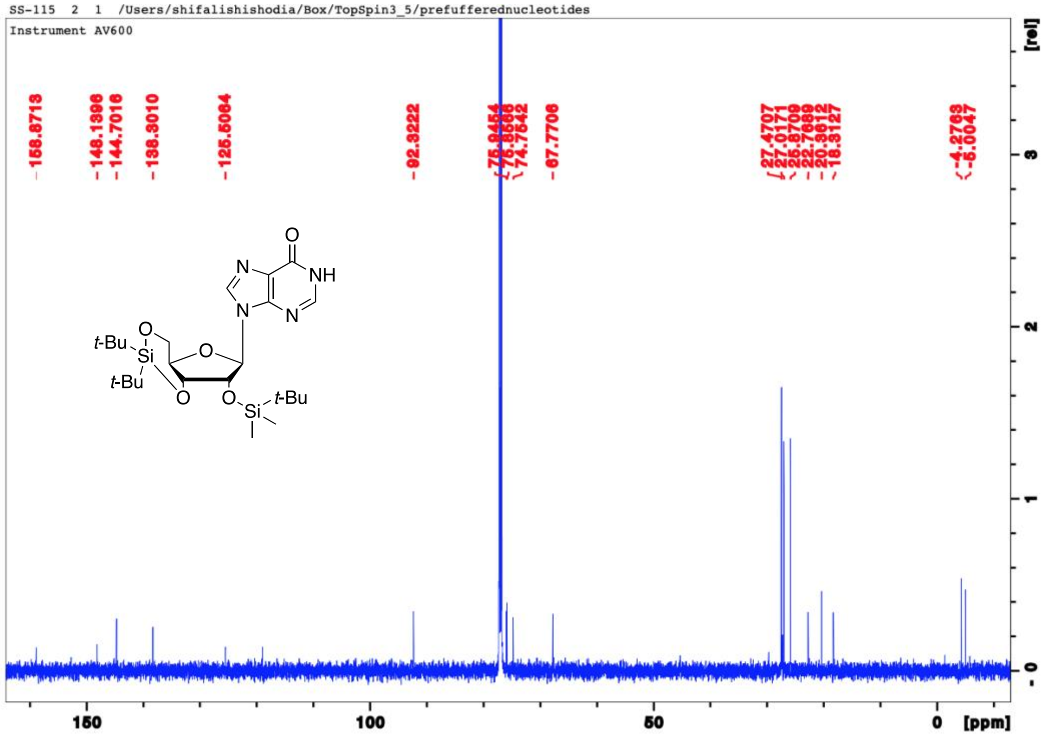

3´,5´-*O*-Bis(*tert*-butyl)silyl-2´-*O*-(*tert*-butyldimethyl)silyl-*N*⁶-methyladenosine (3)

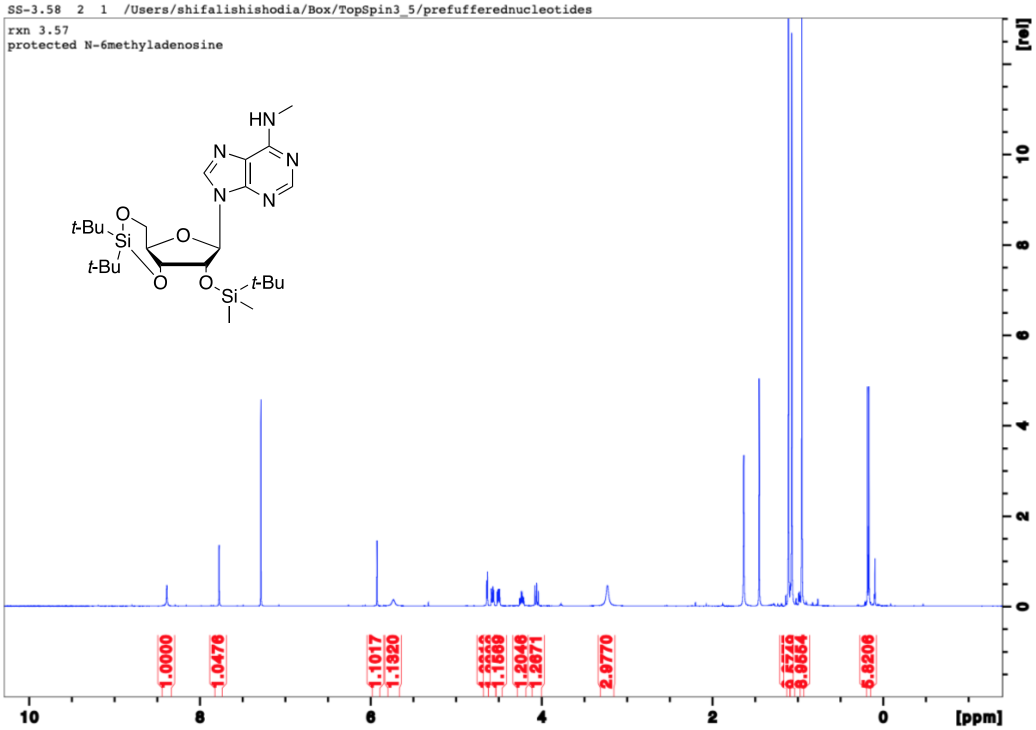

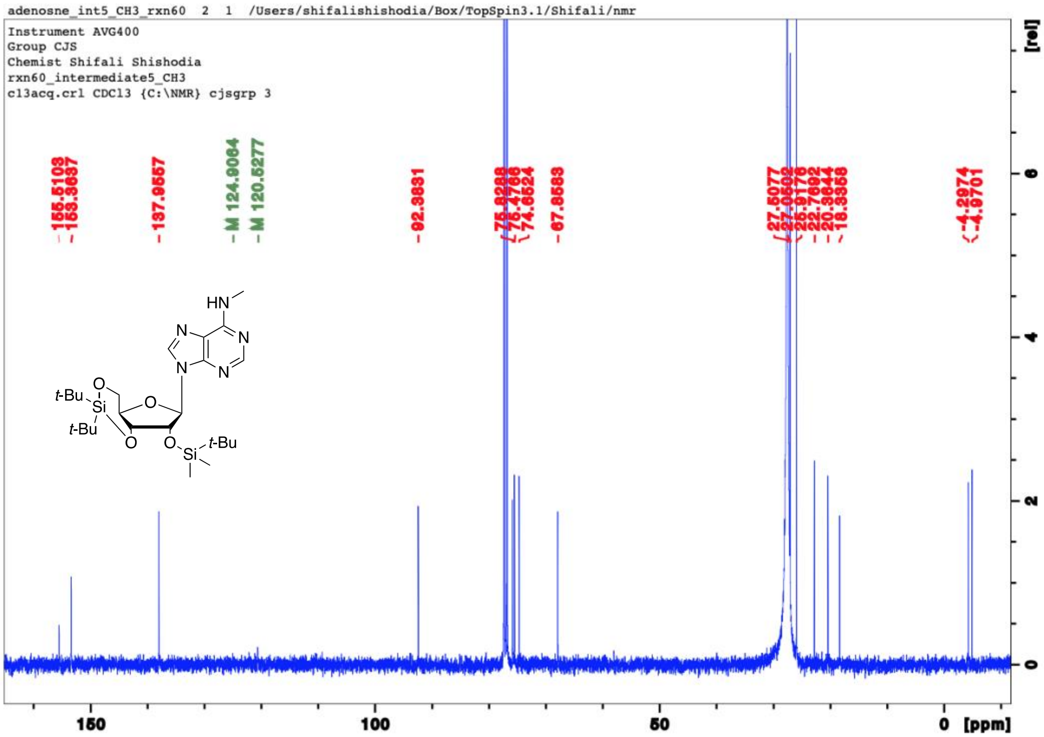

2´-*O*-(*tert*-Butyldimethyl)silyl-*N*[6]-methyladenosine (4)

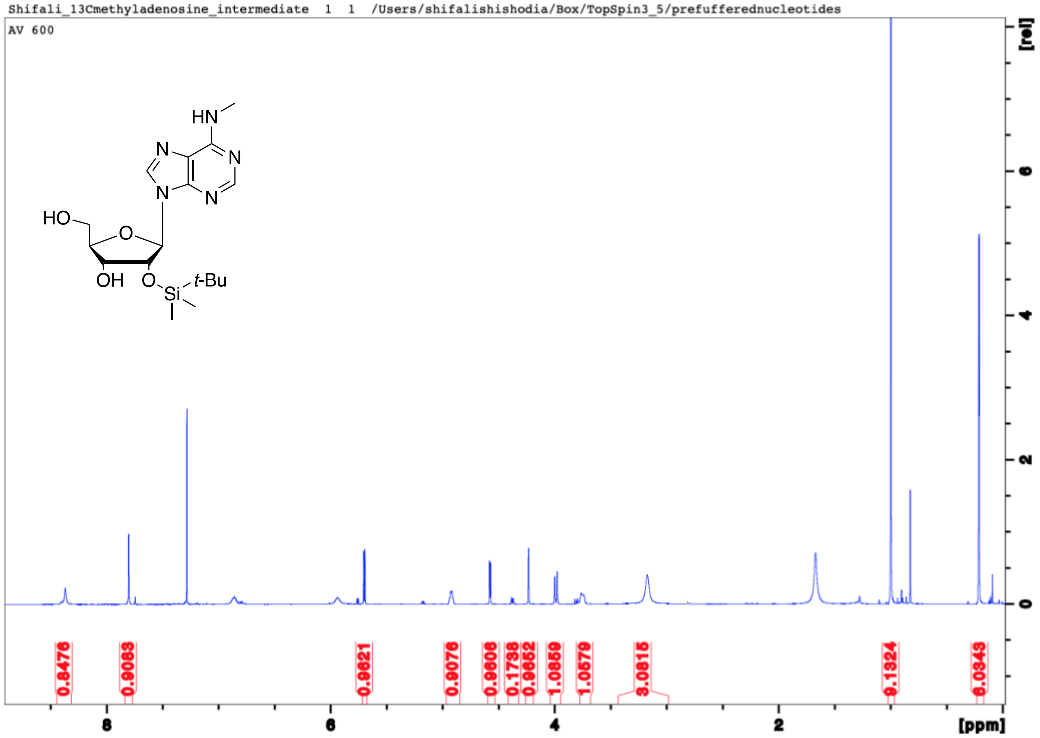

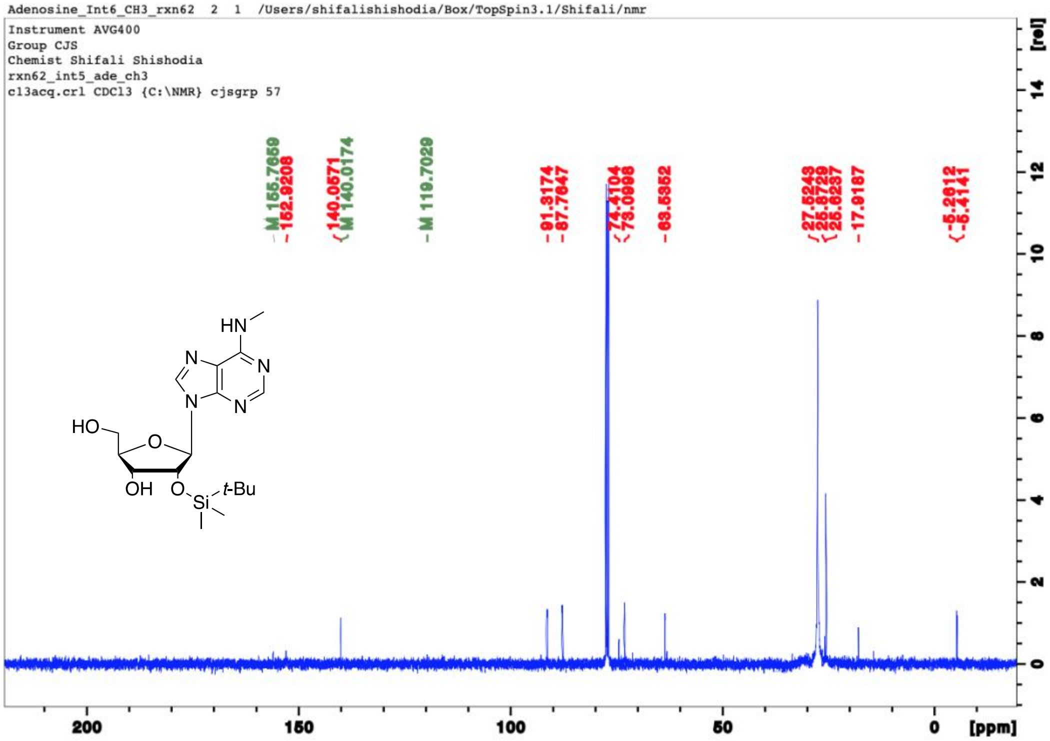

5′-*O*-(4,4′-Dimethoxytrityl)-2′-*O*-dimethyl(*tert*-butyl)silyl-*N*6-methyladenosine (5)

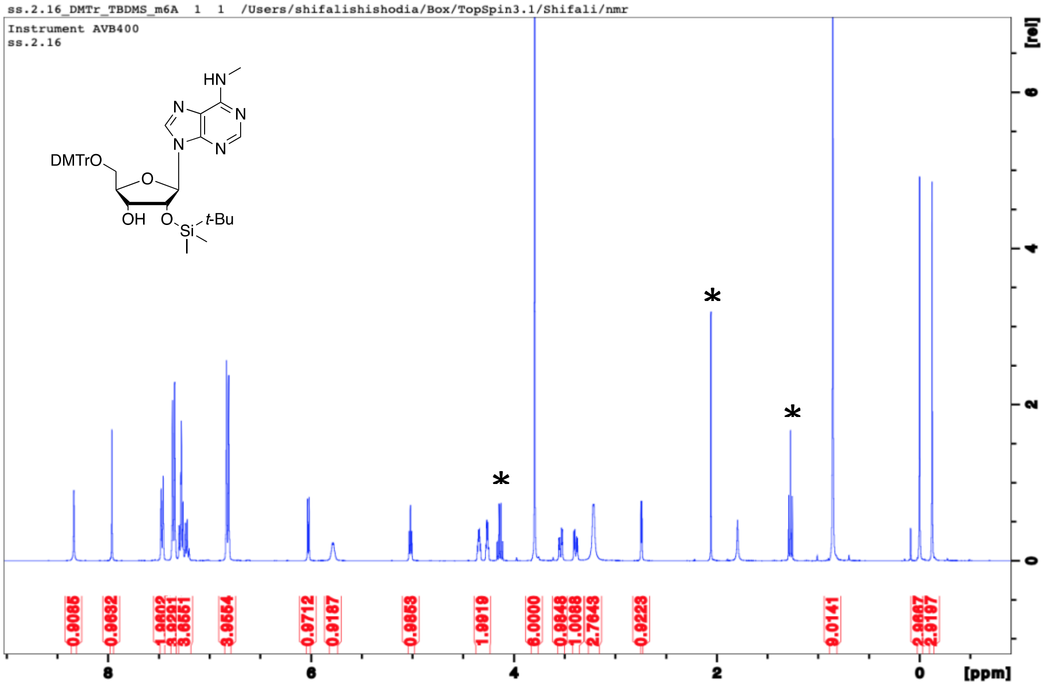

* solvent peaks (ethyl acetate)

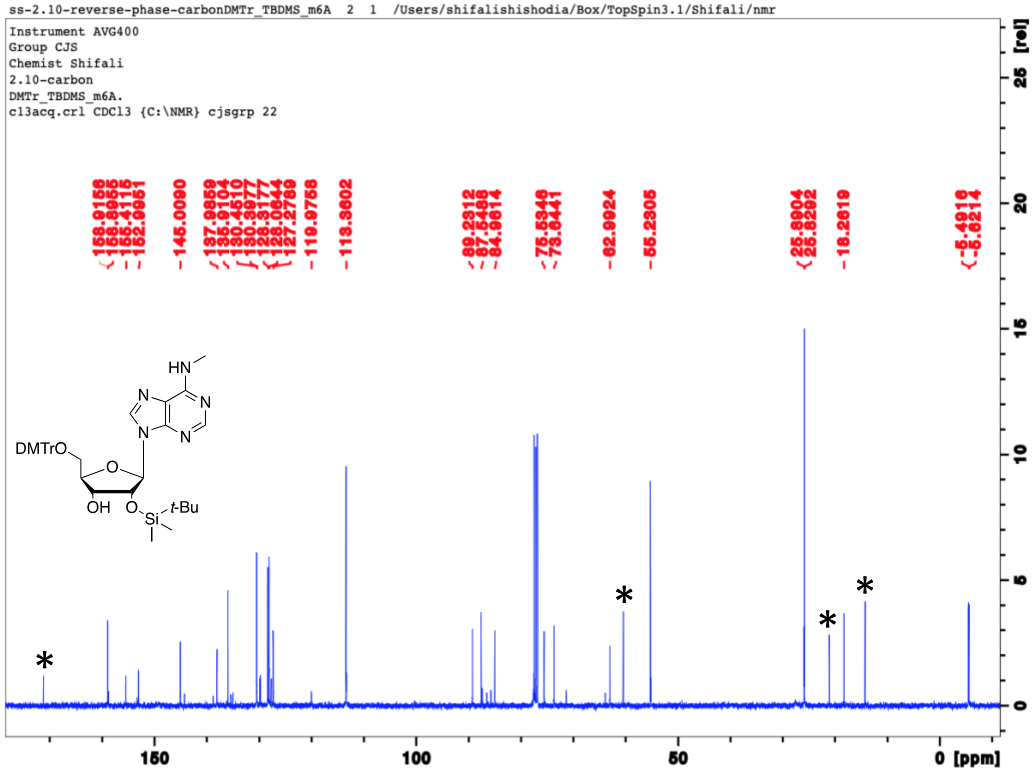

* solvent peaks (ethyl acetate)

5′-*O*-(4,4′-Dimethoxytrityl)-(3′-*O*-[(2cyanoethyl)(*N*,*N*-diisopropylamino)phosphino]-2′-*O*-dimethyl(*tert*-butyl)silyl-*N*[6]-methyladenosine (6)

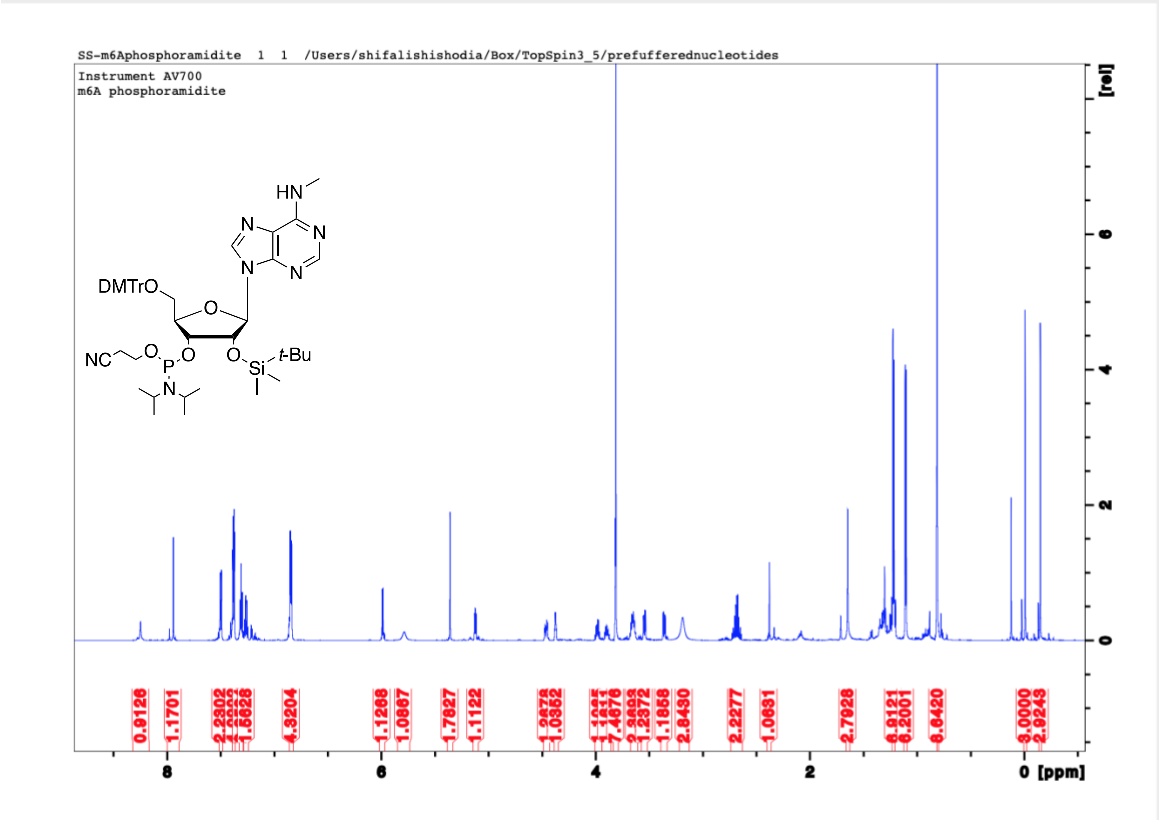

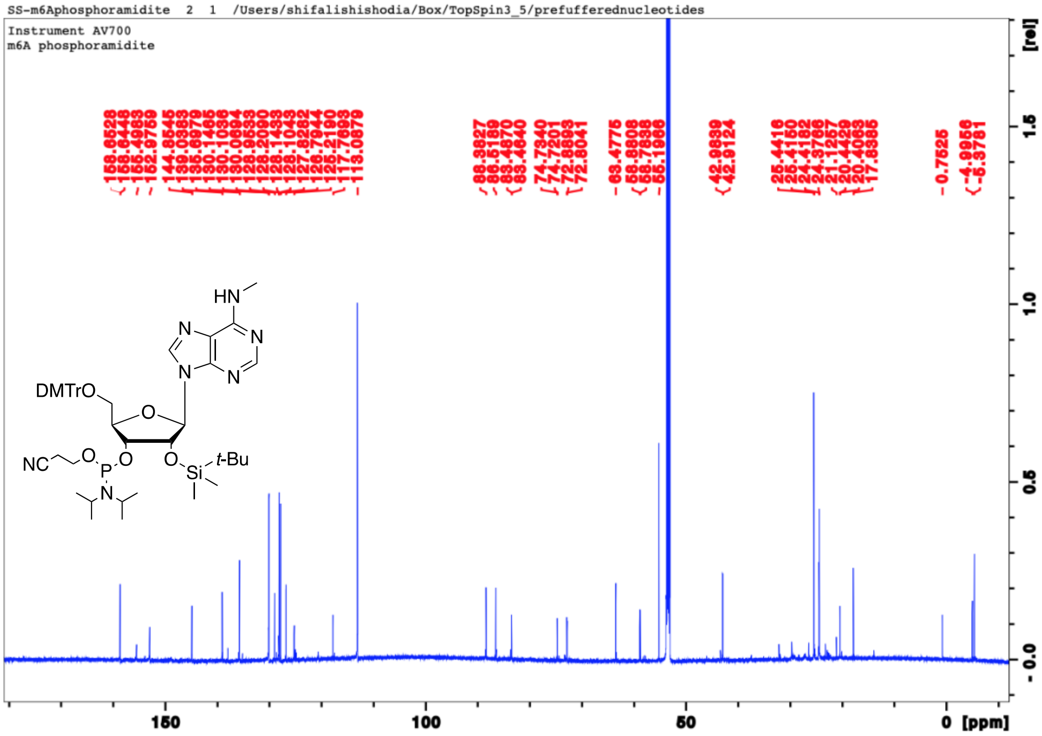

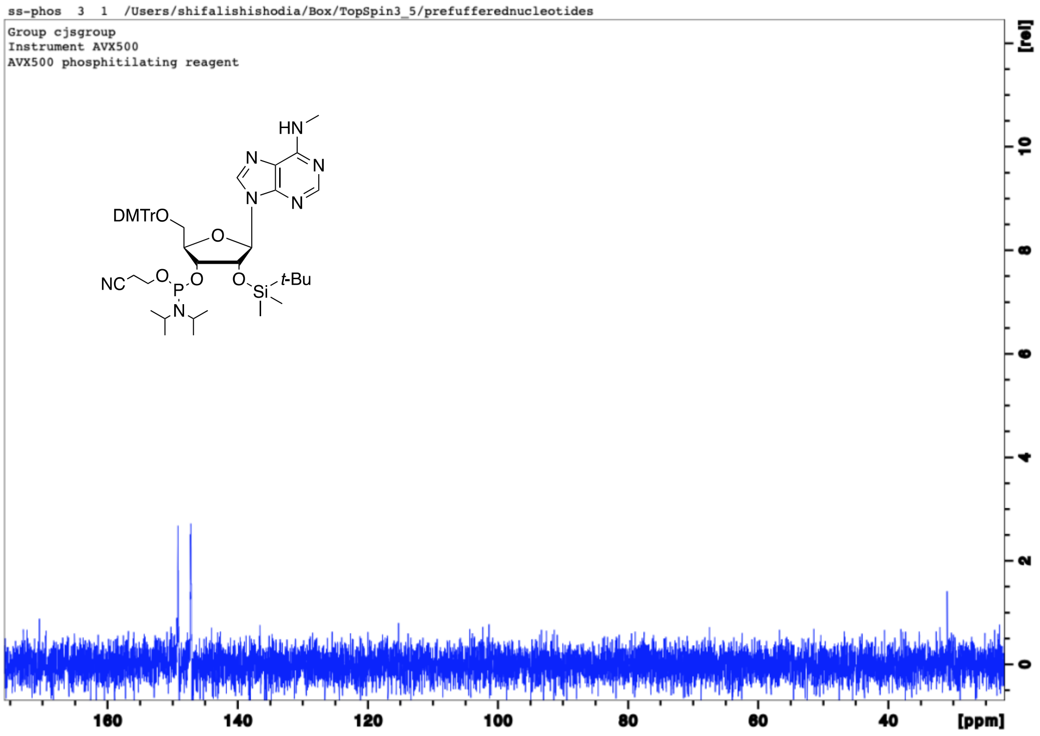
ss-phos 3 1 /Users/shifalishishodia/Box/TopSpin3_5/prefufferednucleotides
Group cjsgroup
Instrument AVX500
AVX500 phosphitilating reagent
HN
N
N
N
N
DMTrO
O
NC
O
P
O
N
O
Si
t-Bu
[rel]
10
8
6
4
2
0
160
140
120
100
80
60
40
[ppm]

N^6-Methyladenosine (1)

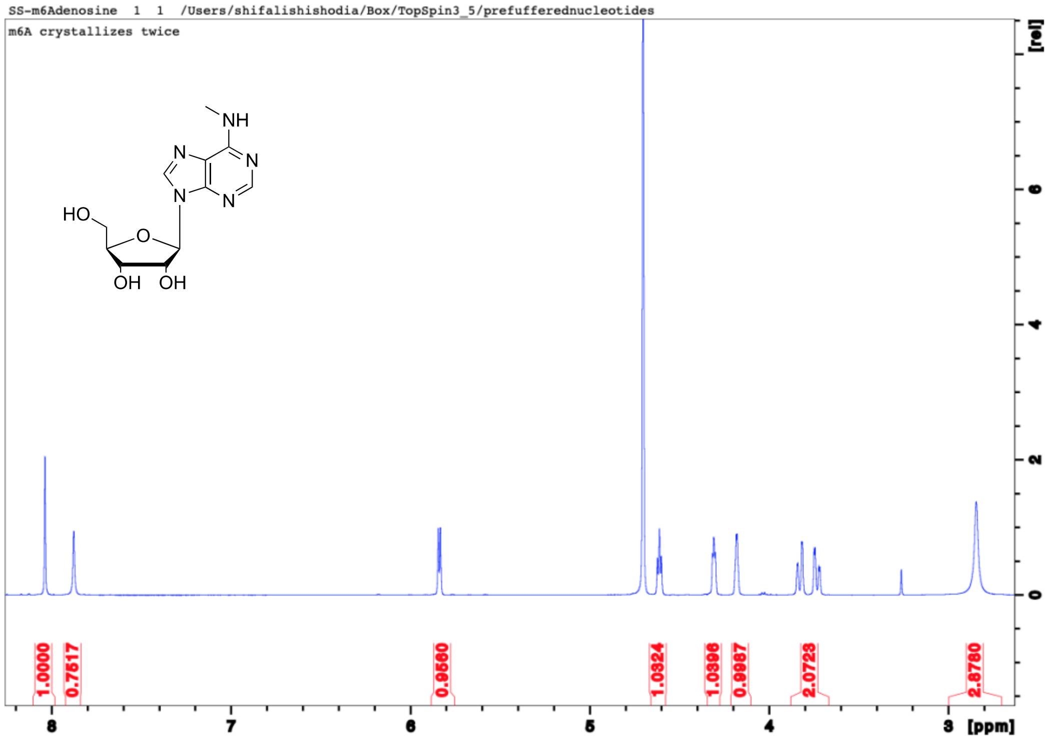

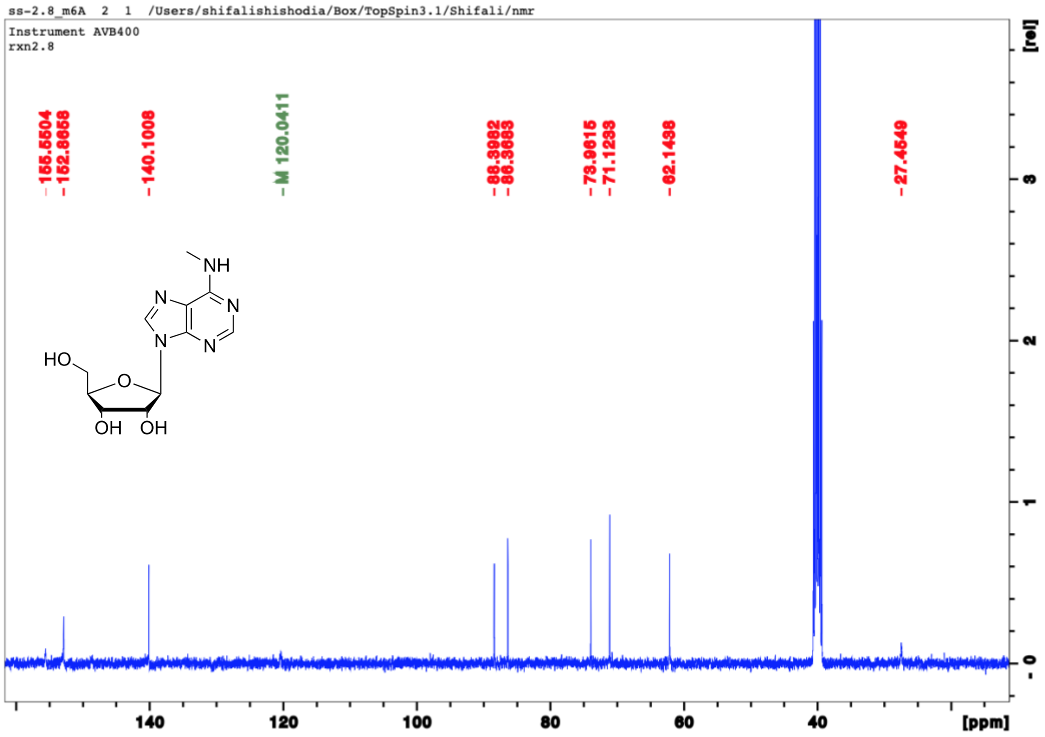

***N*[6], *N*[6]-Dimethyladenosine (11a)**

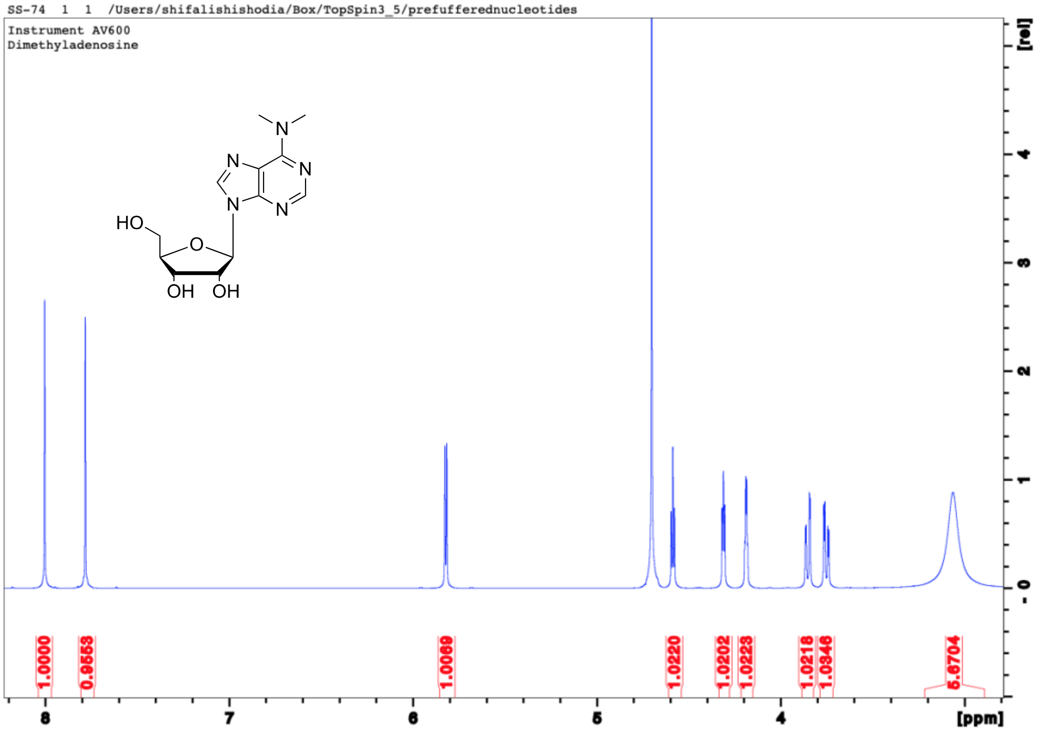

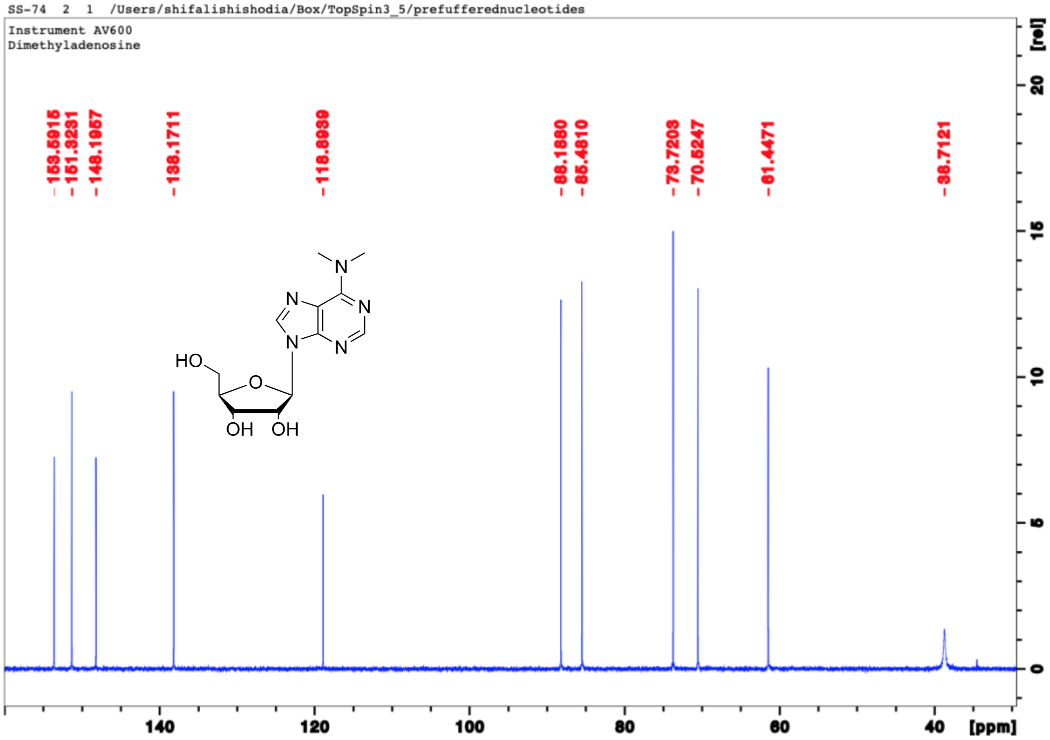

***N*[6]-Ethyladenosine (11b)**

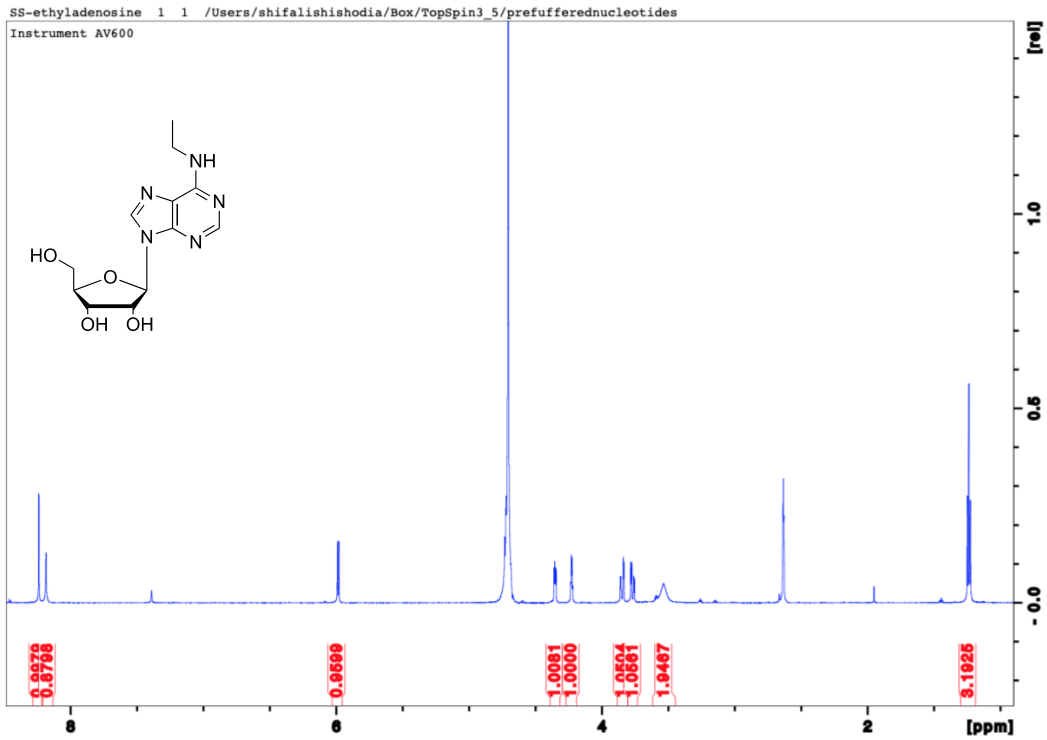

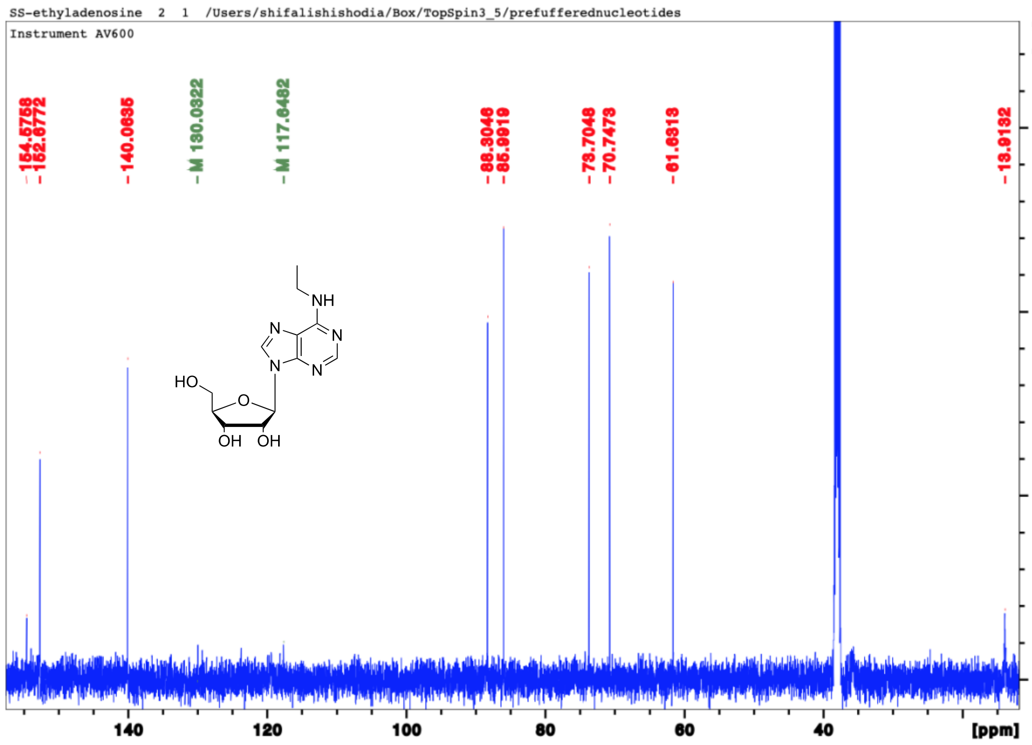

N^6-Cyclopropyladenosine (11c)

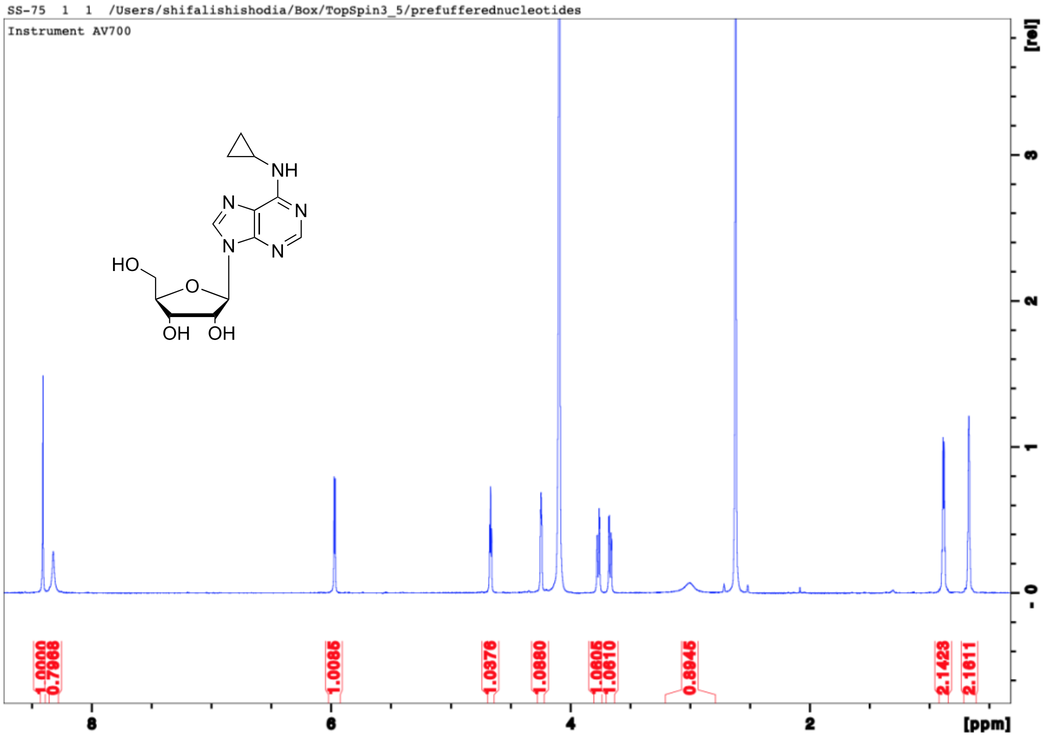

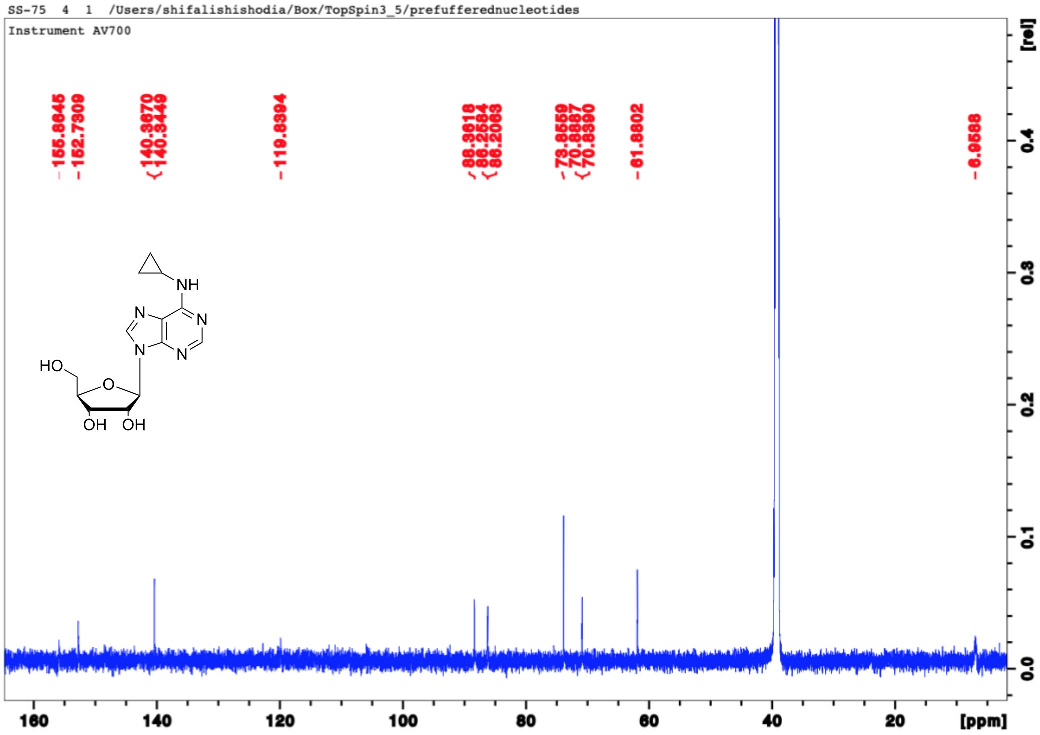

O^6-Methylinosine (11d)

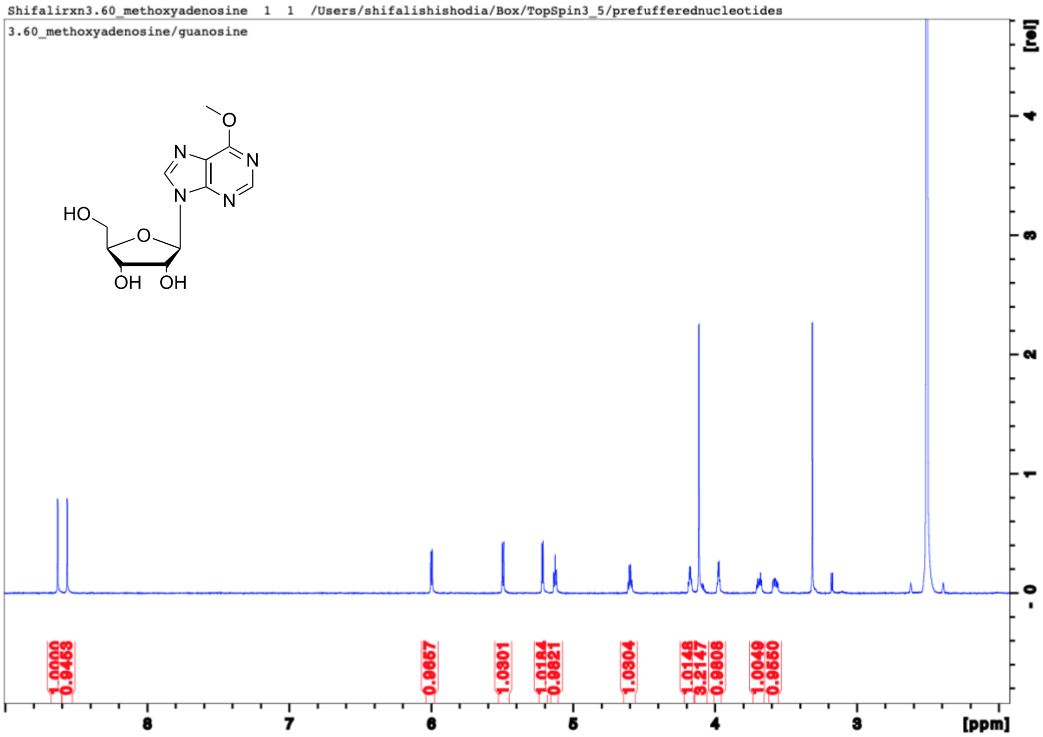

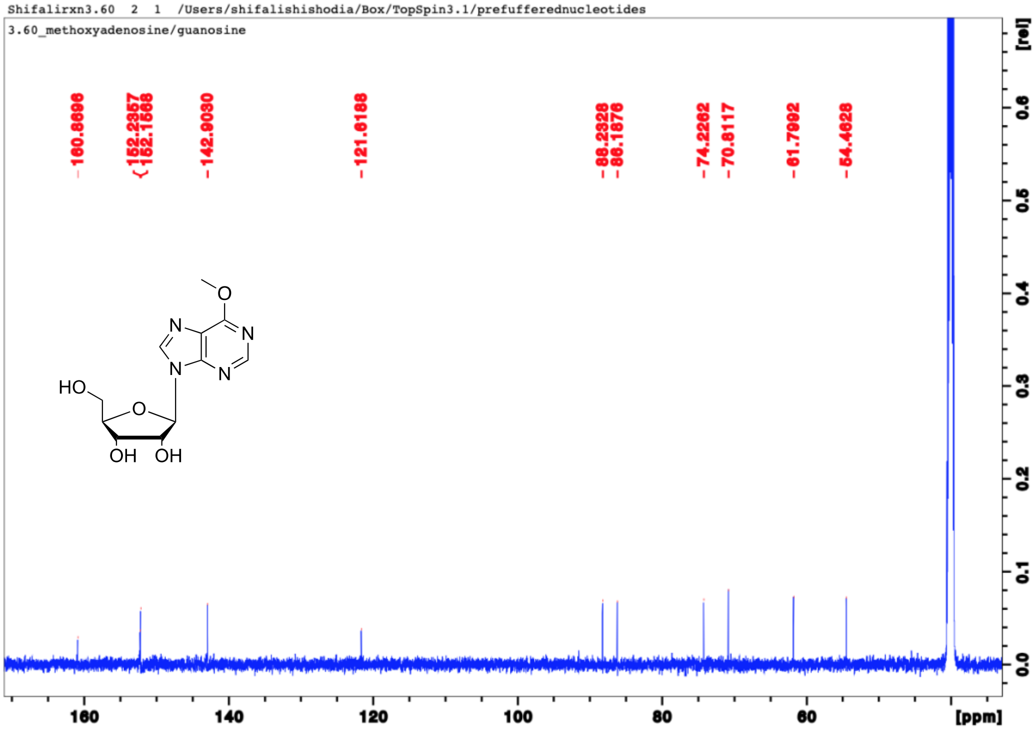

N^6-(2-Hydroxyethyl)adenosine (11e)

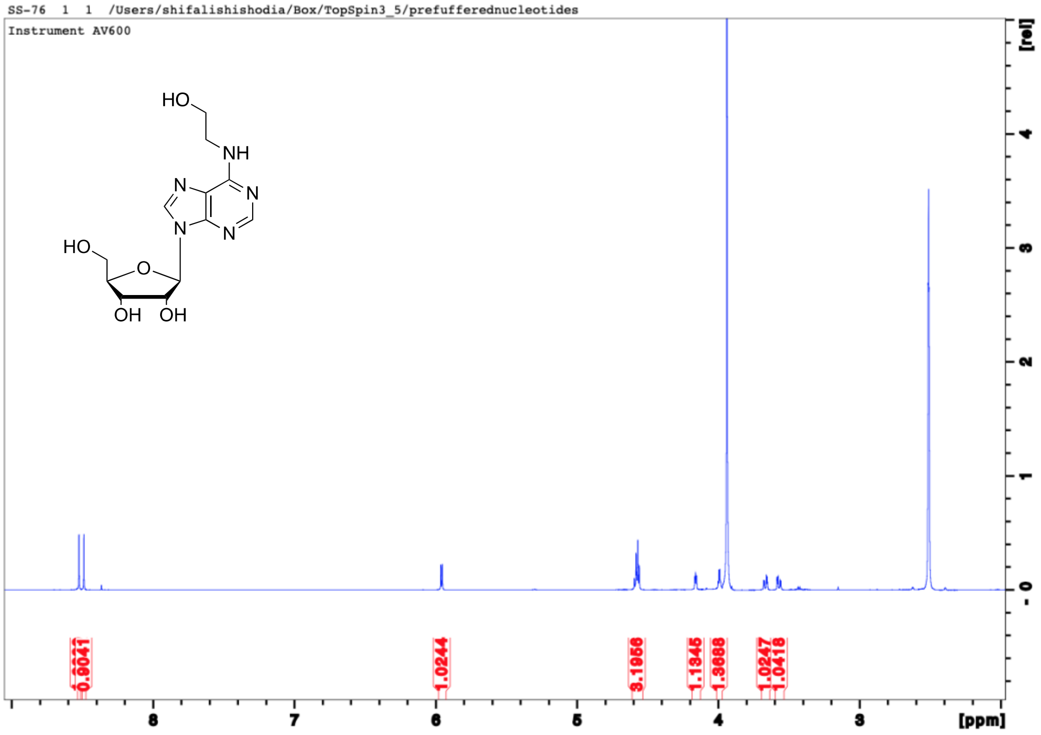

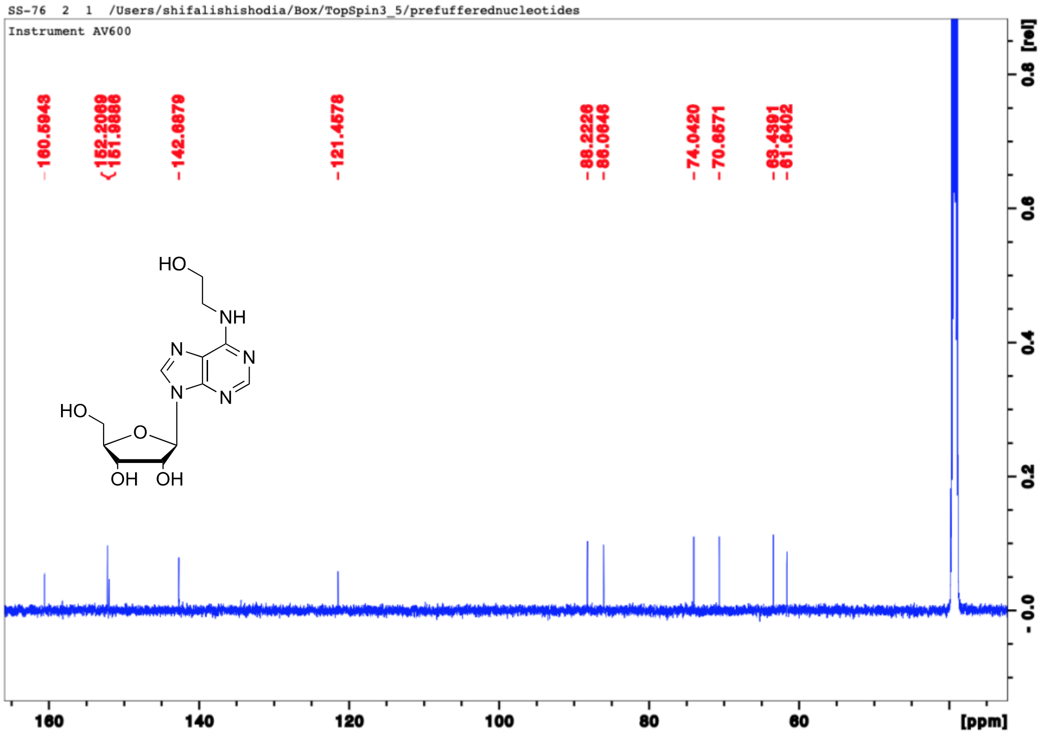

***N*[6],*N*[6]-Methyl(2-hydroxyethyl)adenosine (11f)**

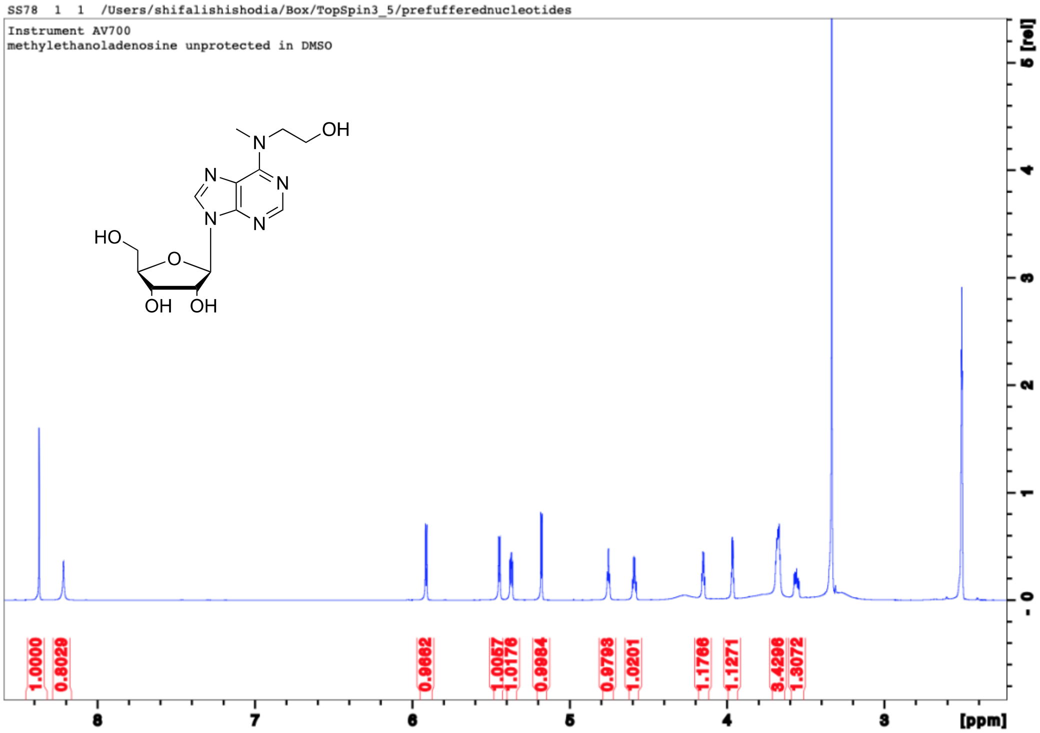

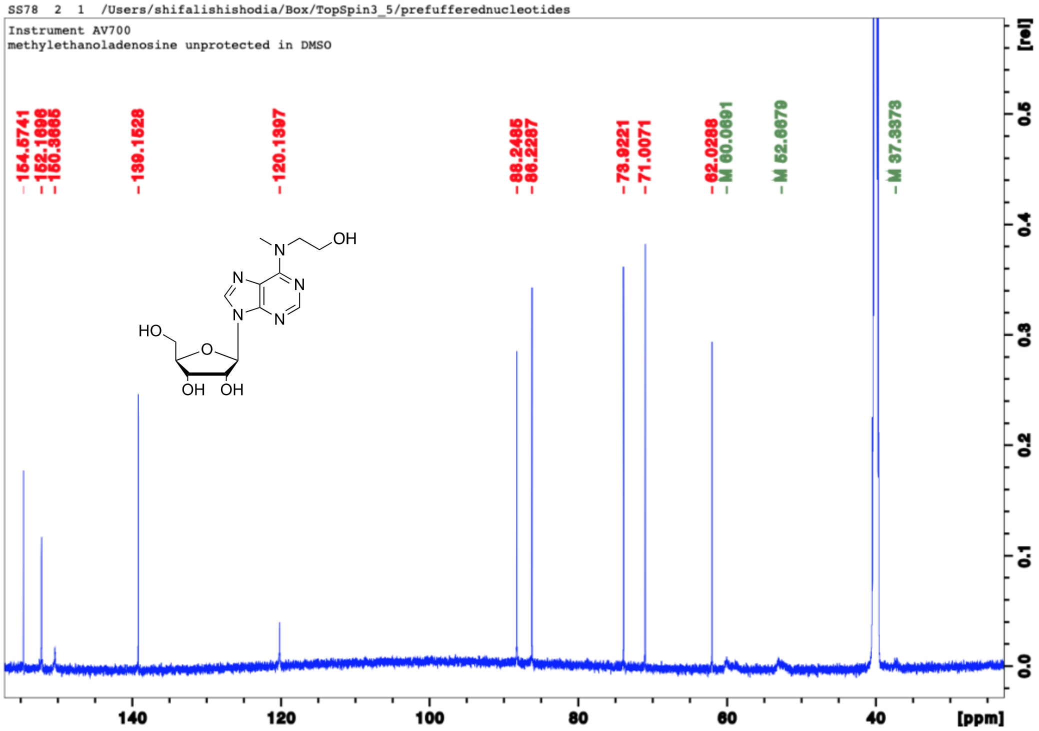

***N*[6],*N*[6]-Ethyl(2-hydroxyethyl)adenosine (11g)**

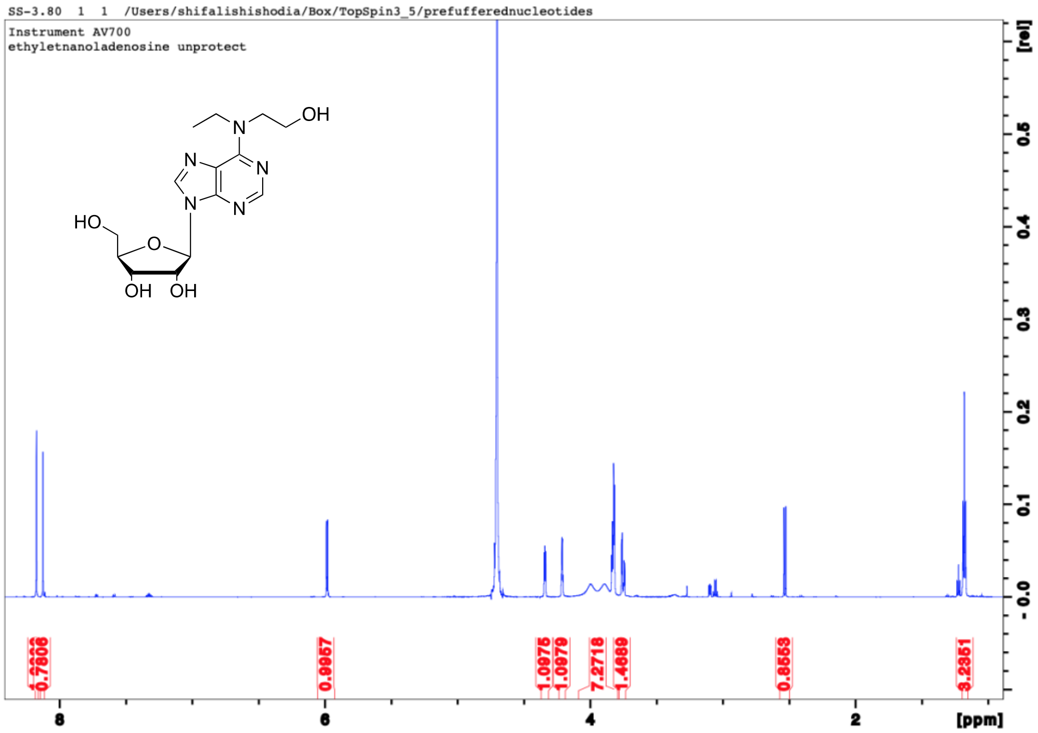

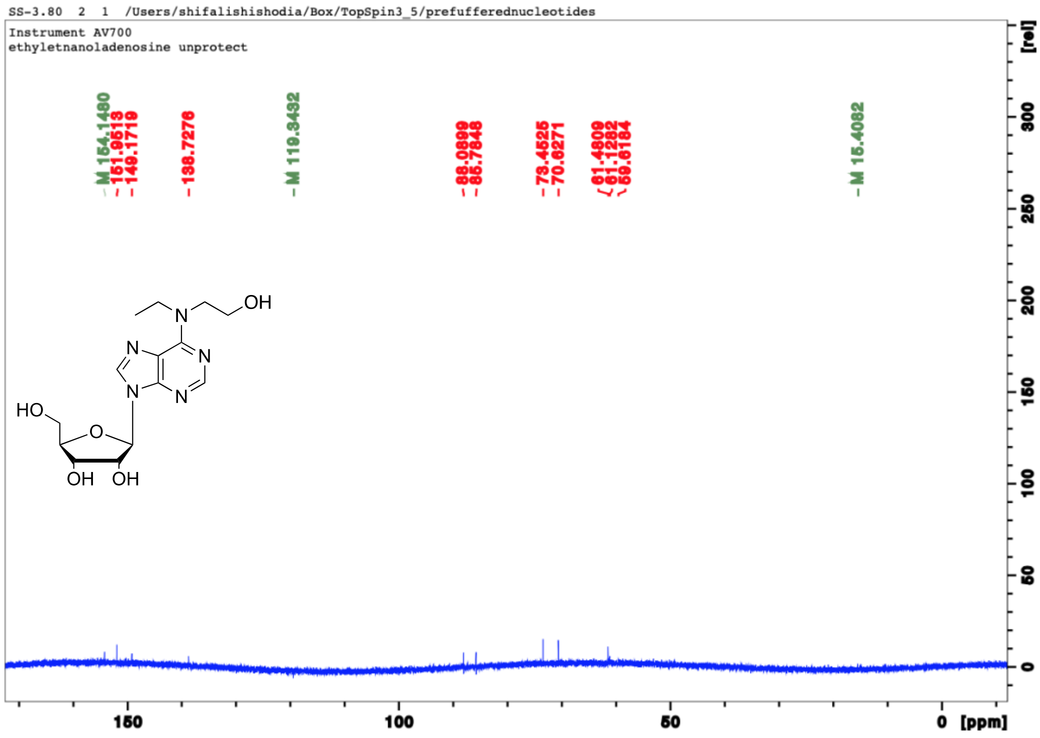

2´,3´,5´-*O*-Tris(*tert*-butyldimethyl)silylinosine (12)

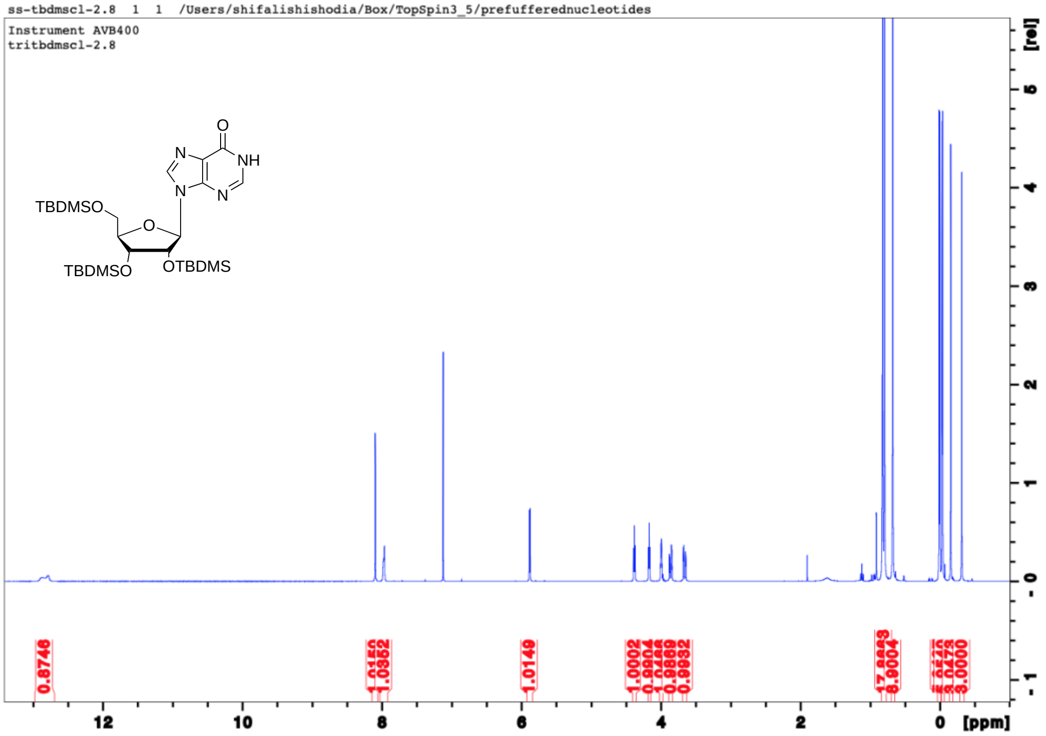

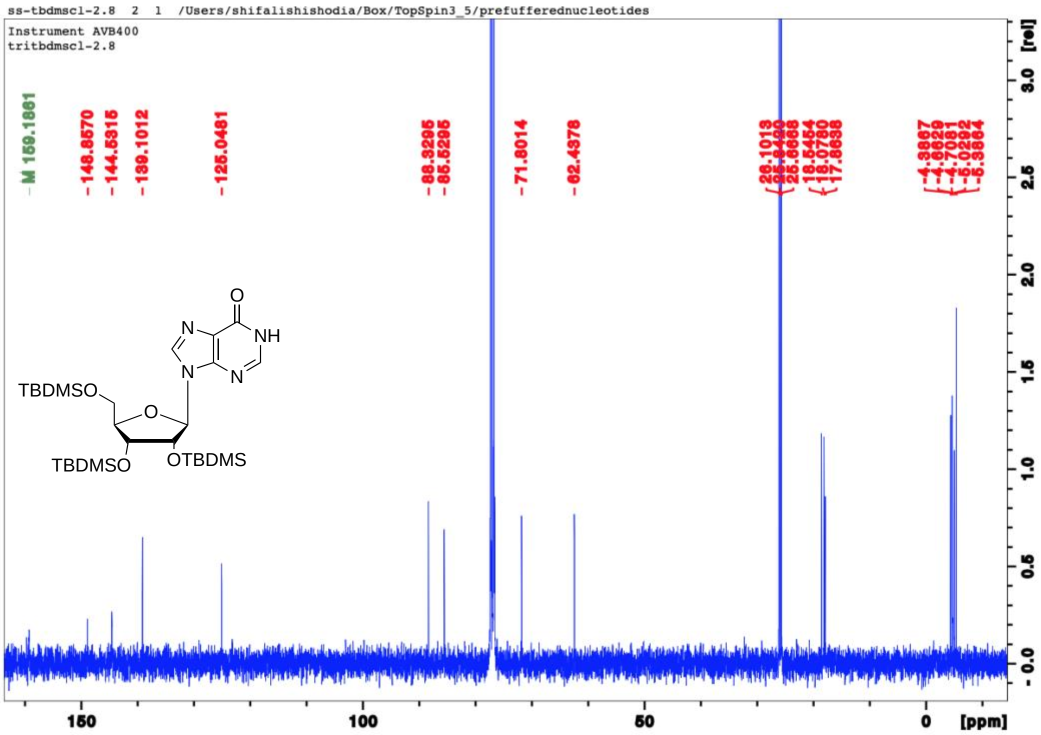

2´,3´,5´-*O*-Tris[dimethyl(*tert*-butyl)silyl]-*N*[6]-(2-hydroxyethyl)adenosine (13a)

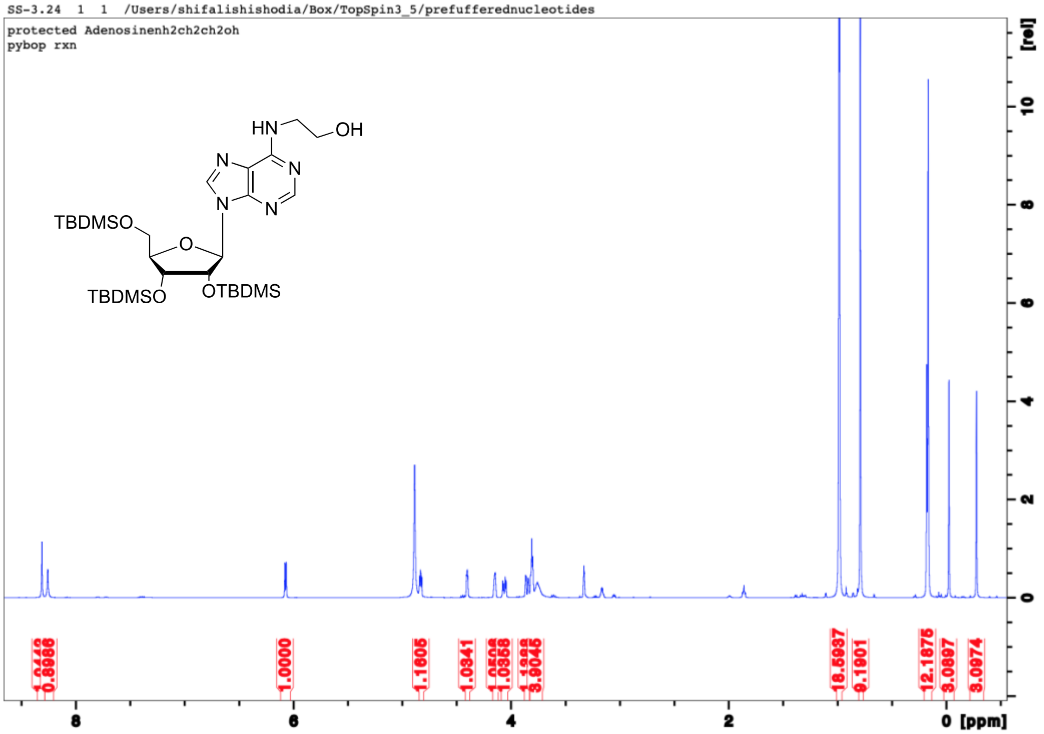

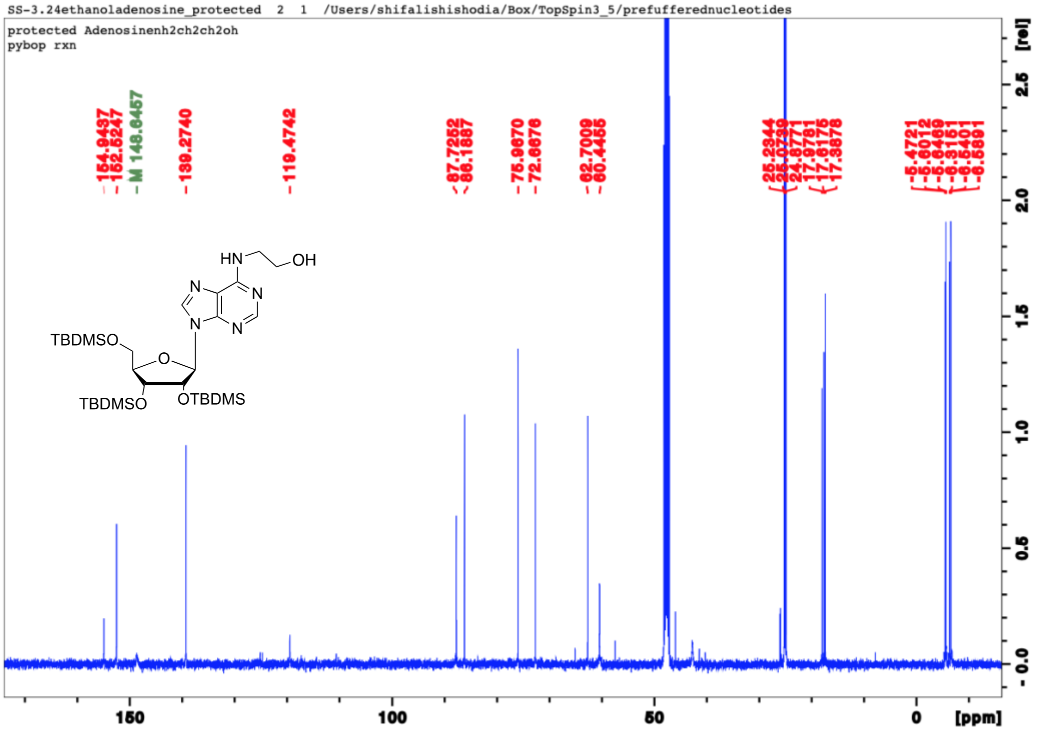

2´,3´,5´-*O*-Tris[dimethyl(*tert*-butyl)silyl]-*N*⁶,*N*⁶-methyl(2-hydroxyethyl)adenosine (13b)

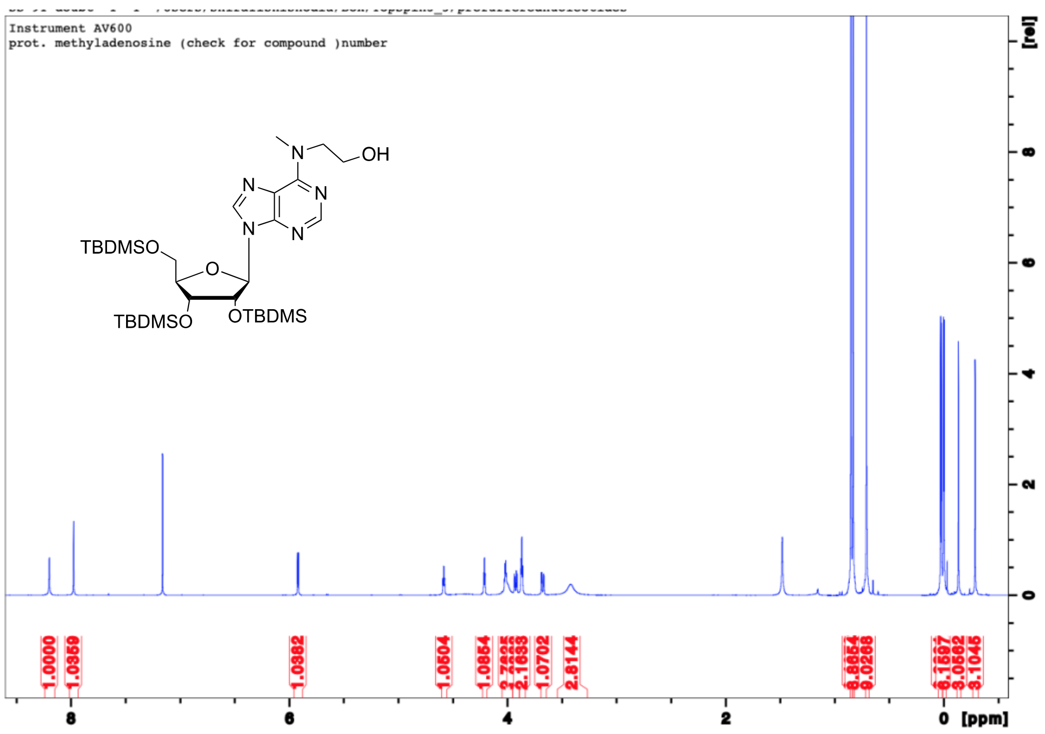

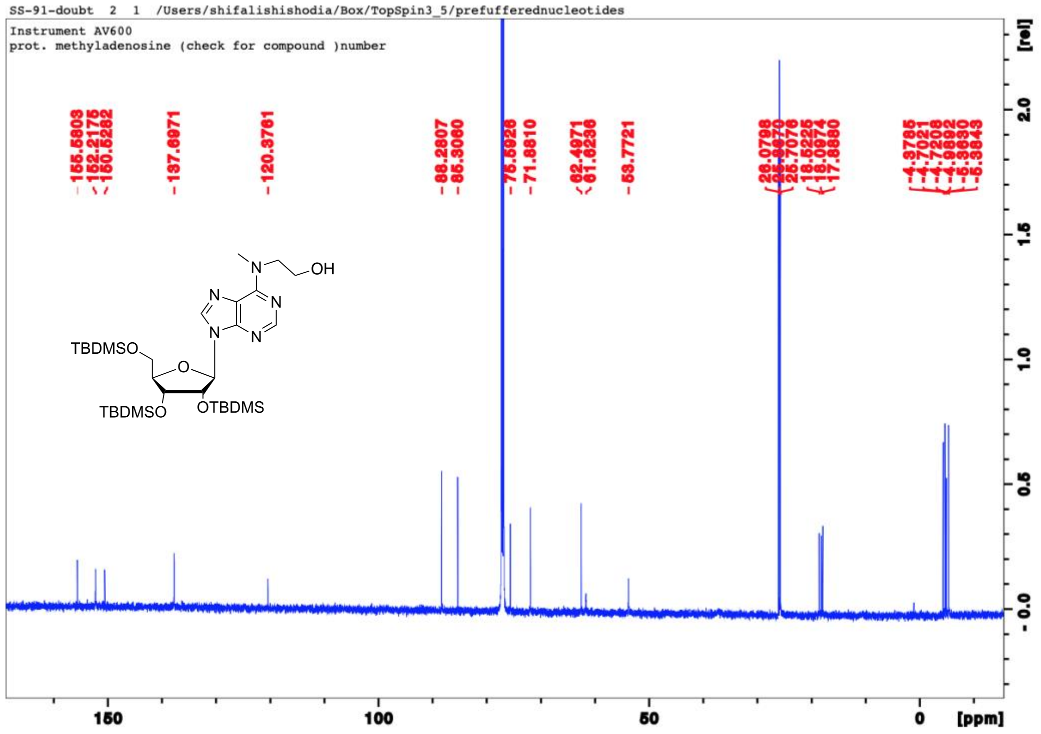

2´,3´,5´-*O*-Tris[dimethyl(*tert*-butyl)silyl]-*N*[6],*N*[6]-ethyl(2-hydroxyethyl)adenosine (13c)

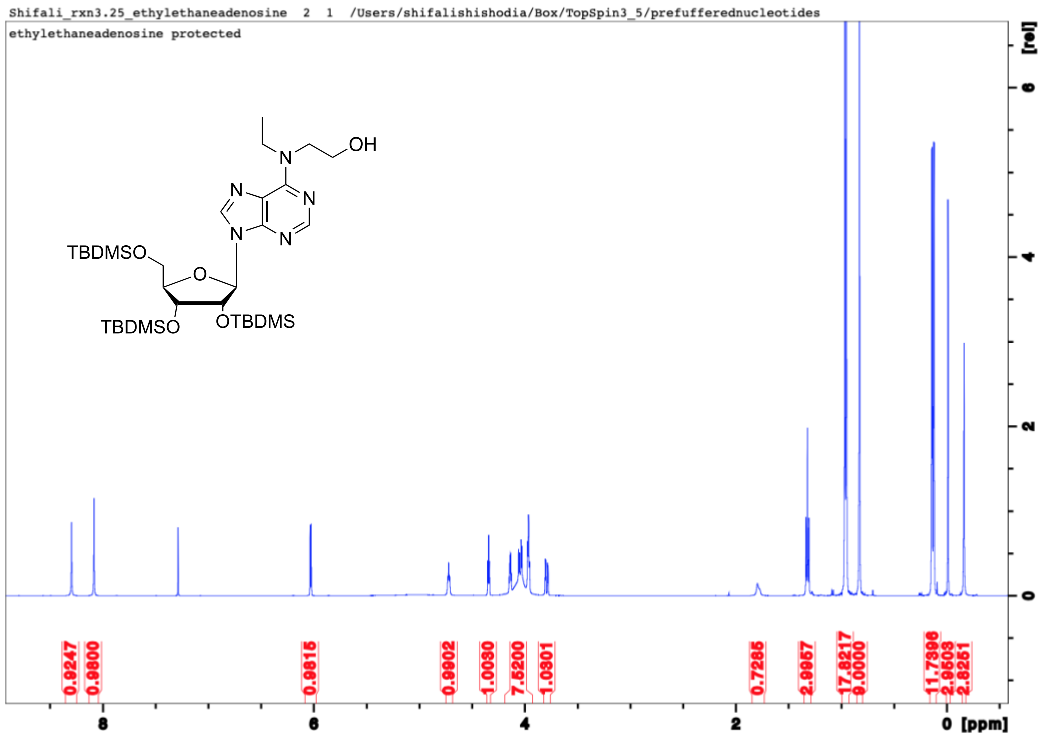

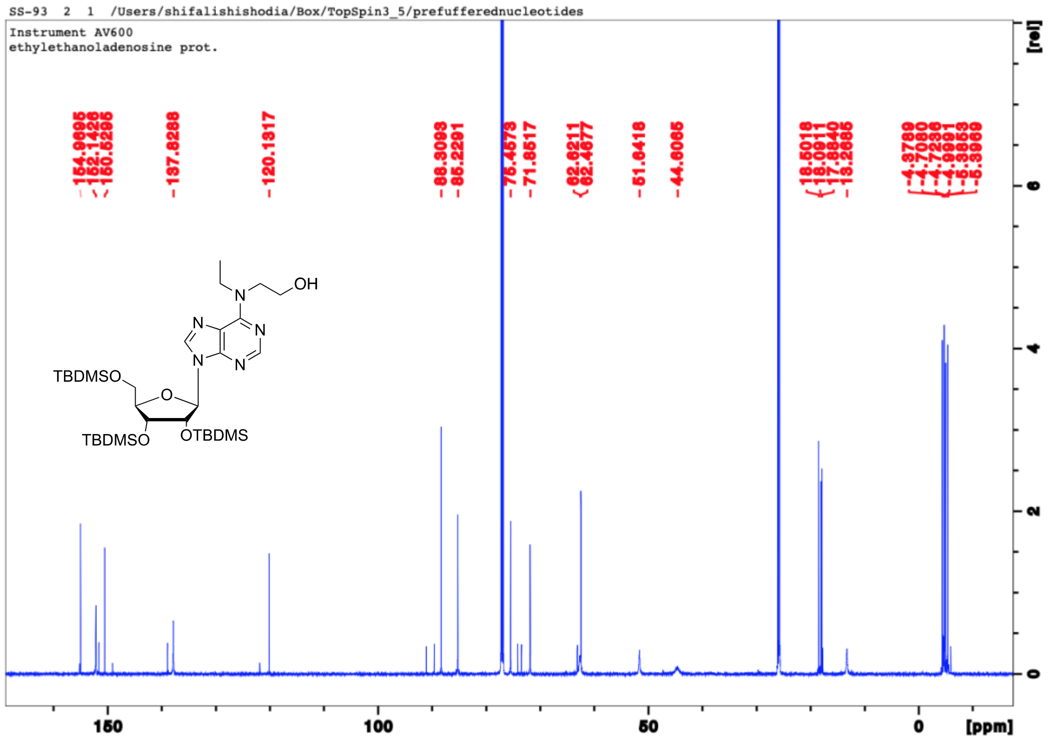

2´,3´,5´-*O*-Tris[dimethyl(*tert*-butyl)silyl]-*N*[1],*N*[6]-ethanoadenosine (15a)

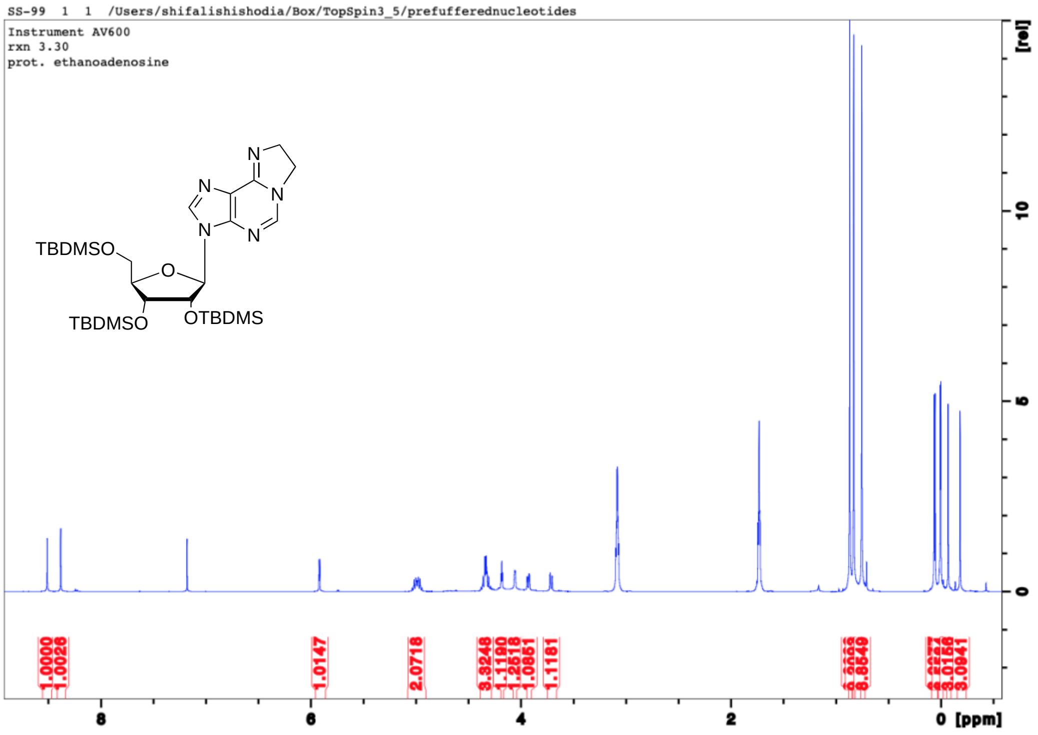

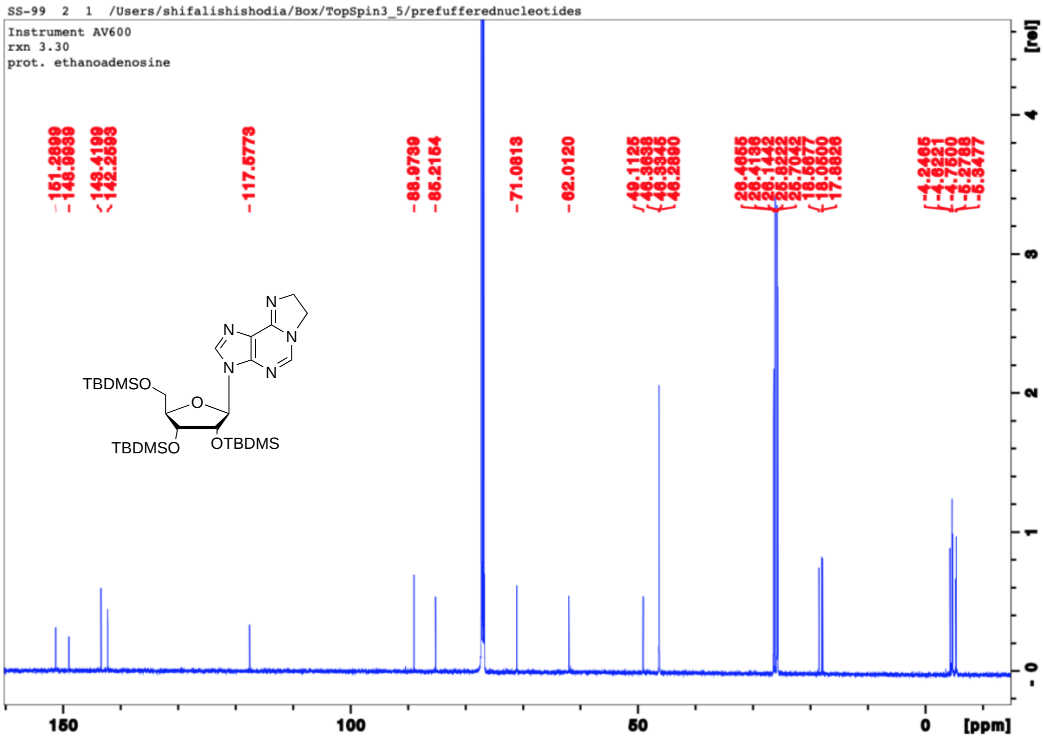

2´,3´,5´-*O*-Tris[dimethyl(*tert*-butyl)silyl]-*N*[6],*N*[1],*N*[6]-methylethanoadenosine (15b)

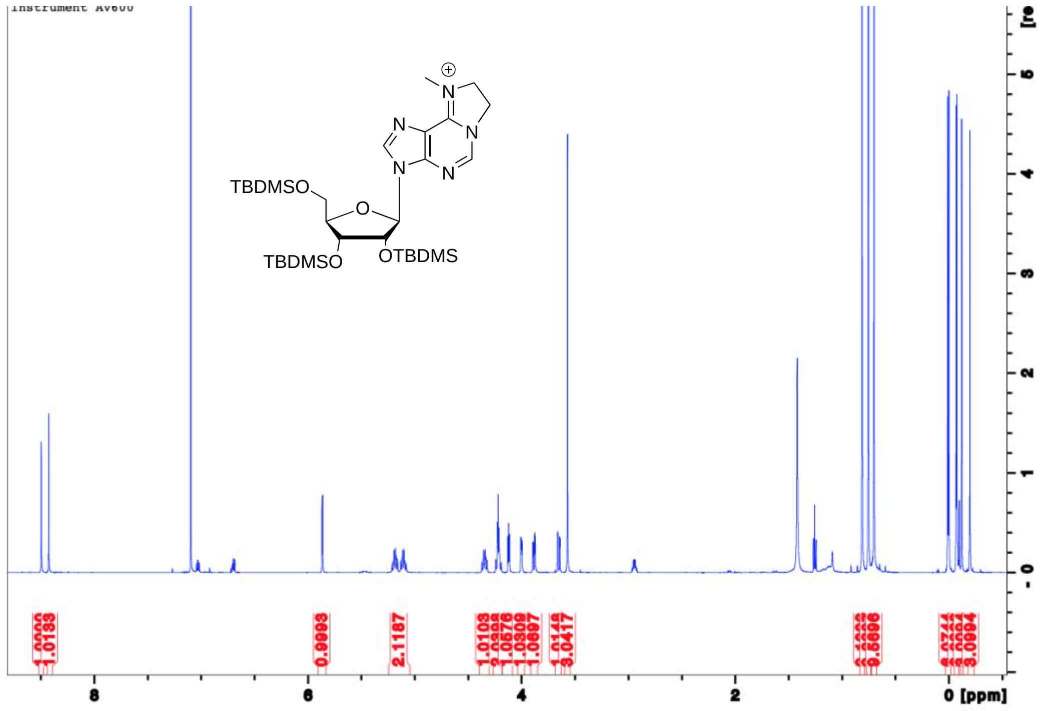

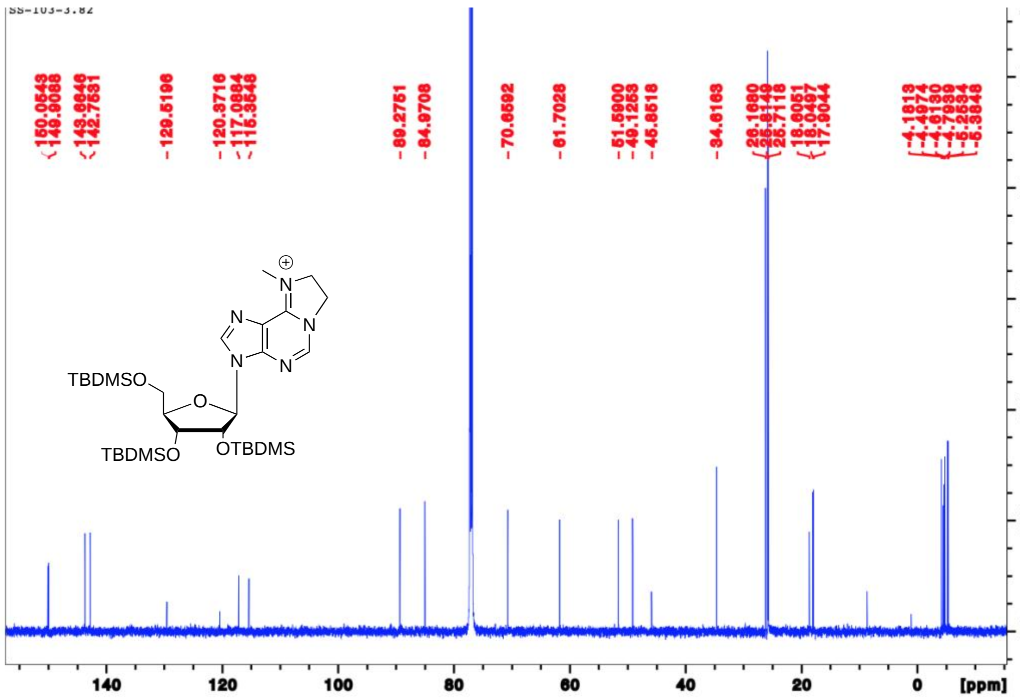

***N*[1],*N*[6]-Ethanoadenosine (16a)**.

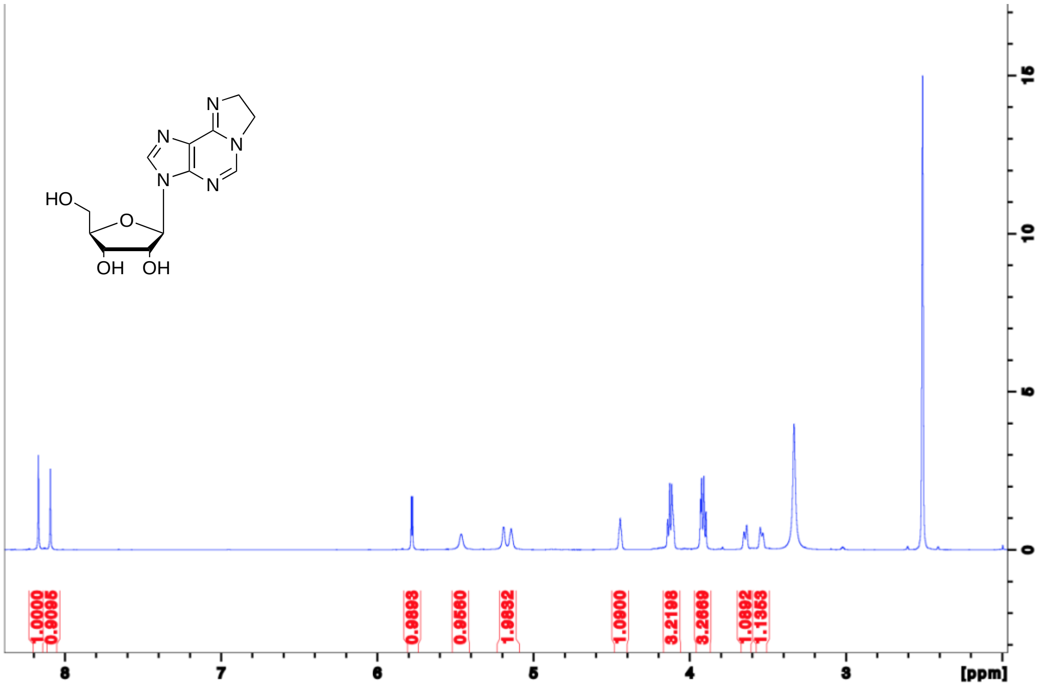

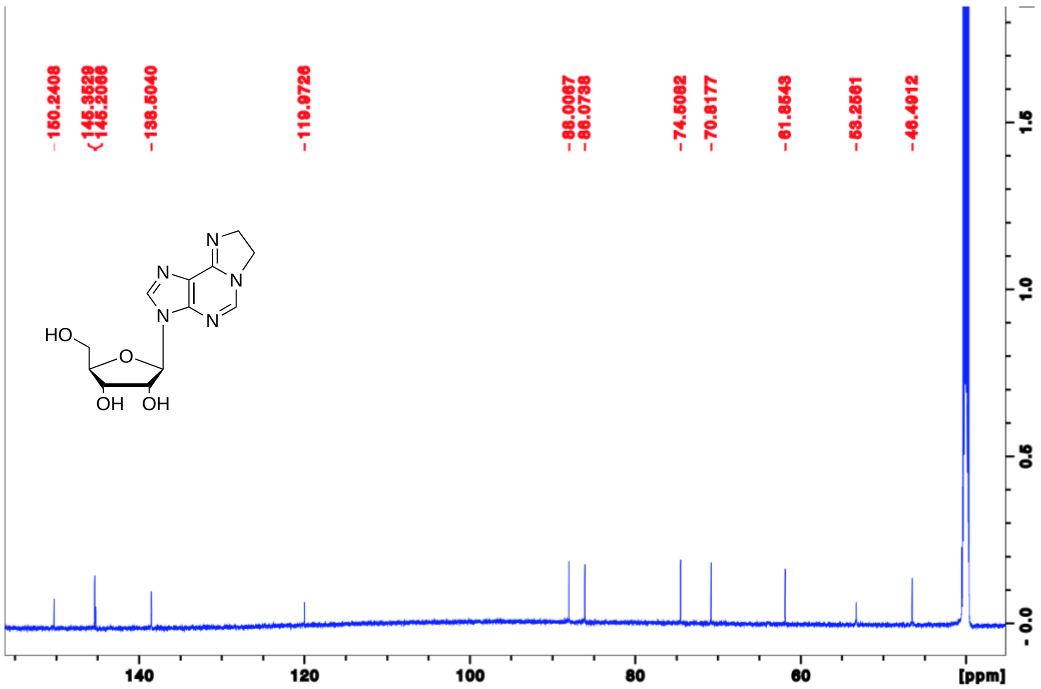

***N*[6],*N*[1],*N*[6]-Methylethanoadenosine (16b)**

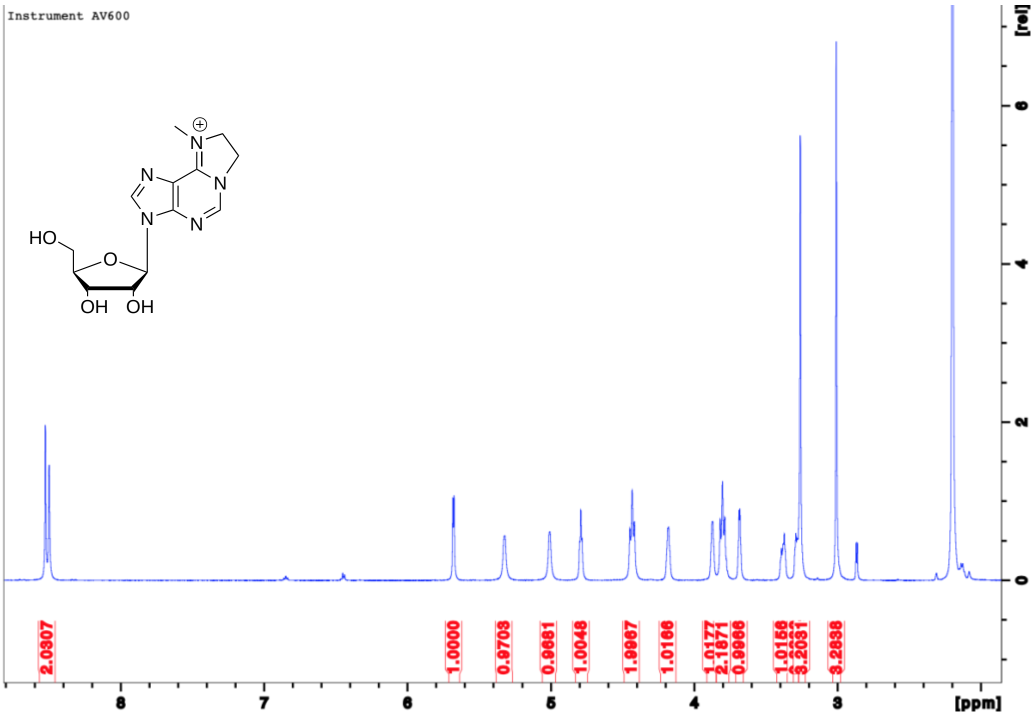

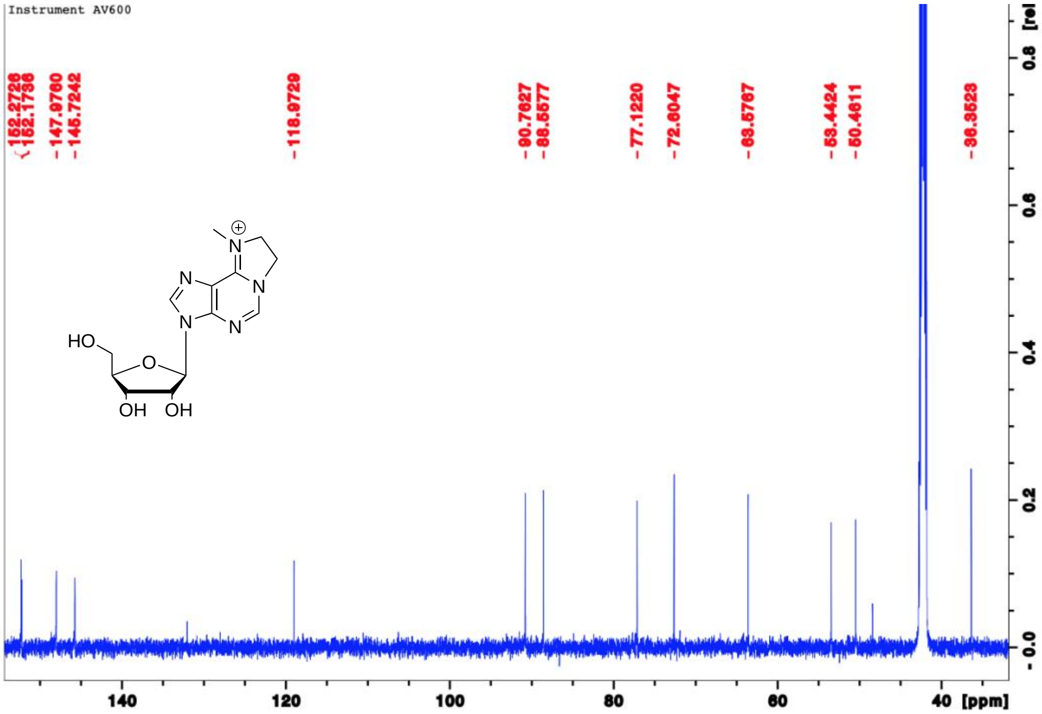

Supplement: Supplementary file 1 [file molecules-26-00147-s001.pdf]
